# ASO Author Reflections: Is It Safe to Use Intrauterine Manipulators in Laparoscopic Surgery for Endometrial Cancer?

**DOI:** 10.1245/s10434-022-12484-2

**Published:** 2022-08-26

**Authors:** Franziska Siegenthaler, Silke Johann, Michael D. Mueller

**Affiliations:** 1 Department of Obstetrics and Gynecology, Bern University Hospital, University of Bern, Bern, Switzerland; 2 Department of Obstetrics and Gynecology Spitalzentrum Oberwallis, Standort Visp, Visp, Switzerland

## Past

Endometrial cancer, the most common gynecologic tumor in developed countries, has a generally favorable prognosis, with an overall 5-year survival rate of 80%. Its primary treatment consists of surgery including total hysterectomy and bilateral salpingo-oophorectomy as well as nodal staging if indicated.^1^ Currently, minimally invasive surgery is the standard approach in early-stage endometrial cancer according to evidence showing no compromise in oncologic outcomes but lower morbidity and a shorter hospital stay than with open surgery.^2^ However, only limited data on the oncological safety of the use of intrauterine manipulators are available. There is some evidence that intrauterine manipulation may result in retrograde seeding of the peritoneal cavity with cancer cells,^3^ and a recently published retrospective trial analyzing 2661 patients showed an association of the use of intrauterine manipulators with higher recurrence rates and worse survival in endometrial cancer.^4^ This study aimed to analyze the association of intrauterine manipulation, peritoneal cytology, and oncologic outcomes for endometrial cancer patients.

## Present

This multicenter prospective trial included 124 endometrial cancer patients undergoing laparoscopic staging surgery with the use of an intrauterine manipulator.^5^ Three different sets of peritoneal washings were obtained: at the beginning of the surgical procedure, after insertion of the intrauterine manipulator, and after closure of the vaginal vault. Peritoneal cytology was negative for 98 patients and positive at the beginning of the surgery for 16 patients. During the procedure, 10 patients had a positive cytology conversion. The results showed a strong correlation of recurrence rate with peritoneal cytology, and the patients with converted peritoneal cytology presented with the worst oncologic outcomes. The findings suggest that the use of intrauterine manipulators may lead to a positive peritoneal cytology conversion, which in turn would enhance the recurrence rate. This study supplies crucial knowledge for understanding the impact of the use of intrauterine manipulators on oncologic outcomes for endometrial cancer patients and provides further evidence to fill the remaining gaps between uterine manipulation, peritoneal cytology, and recurrence in endometrial cancer.

## Future

Minimally invasive surgery certainly remains the standard of care in endometrial cancer treatment after the results of prospective randomized trials proving its oncologic safety.^2^ However, these trials did not report on the use of uterine manipulators. Because no data exist to prove that the use of uterine devices reduces surgical complications, and because current evidence shows a safe possibility of performing hysterectomy without the use of intrauterine manipulators, clinicians should consider abandoning intrauterine manipulators in surgery for endometrial cancer. However, larger prospective clinical trials including a control group that has surgery without an intrauterine manipulator are needed to confirm the results of this study.
